# The Effects of the Substrate Length and Cultivation Time on the Physical and Mechanical Properties of Mycelium-Based Cushioning Materials from *Salix psammophila* and Peanut Straw

**DOI:** 10.3390/biomimetics10060371

**Published:** 2025-06-05

**Authors:** Xiaowen Song, Shuoye Chen, Jianxin Wu, Ziyi Cai, Yanfeng Zhang, Risu Na, He Lv, Cong He, Tingting Wu, Xiulun Wang

**Affiliations:** 1 College of Mechanical Engineering, Inner Mongolia University of Technology, Hohhot 010051, China; 2 Inner Mongolia Key Laboratory of Robotics and Intelligent Equipment Technology, Inner Mongolia University of Technology, Hohhot 010051, China; 3 Graduate School of Bioresources, Mie University, 1577 Kurimamachiya-cho, Tsu 5148507, Japan

**Keywords:** lignocellulose, mycelium, mycelium-based cushioning materials, cultivation time, substrate length

## Abstract

Mycelium-based biocomposites represent a novel class of environmentally friendly materials. This study investigated the potential of using *Salix psammophila* and peanut straw as substrates for cultivating *Pleurotus ostreatus* and *Ganoderma lucidum*, respectively, to fabricate mycelium-based cushioning materials. The results demonstrated that the *Pleurotus ostreatus*-based cushion material using *Salix psammophila* (POSM) outperformed the *Ganoderma lucidum*-based cushion material using peanut straw (GLPM) in terms of overall performance. Both materials presented optimal comprehensive properties when the cultivation period reached 30 days. Increasing the substrate length enhanced most of the material properties. The resulting density ranged from 0.13 to 0.16 g/cm^3^, which was higher than that of polystyrene foam. The contact angles of both materials exceeded 120°, whereas their elastic springback rates reached 50.2% and 43.2%, and their thermal conductivities were 0.049 W/m·K and 0.051 W/m·K, respectively. Additionally, thermogravimetric analysis revealed that both materials exhibited similar thermal degradation behavior and relatively high thermal stability. These findings align with those of previous studies on mycelium composites and indicate that the physical and mechanical properties of the materials are largely comparable to those of expanded polystyrene (EPS). In conclusion, the developed mycelium-based cushioning materials promote the efficient utilization of agricultural residues and hold promise as a sustainable alternative to EPS, offering broad application prospects in the transportation and packaging sectors.

## 1. Introduction

As China’s economy continues to expand, the demand for cushioning packaging materials has steadily increased [1]. Currently, commonly used cushion packaging products include foam plastics, corrugated cardboard, honeycomb paper, and molded pulp. These materials protect products from damage during transportation and storage, with foam plastic packaging dominating the market because of its malleability, lightweight nature, corrosion resistance, and affordability [2,3]. Most plastics in use today are petroleum-based and nonbiodegradable, resulting in waste that can persist in the environment for centuries [4]. For example, expanded polystyrene (EPS) is widely used in packaging because of its low density, high stiffness, excellent insulation, and easy molding [5,6,7]. Conventional disposal methods such as incineration and landfilling pose significant environmental challenges. EPS degrades very slowly in nature and can leach toxic chemicals [8]. Incineration in particular produces smoke laden with carcinogenic hydrocarbons that threaten human and animal health [9]. At current consumption rates, global oil reserves may be depleted within 50–60 years [10]. Many countries now regulate the use of polyethylene (PE), polypropylene (PP), and polystyrene (PS) foams and promote biodegradable alternatives [11,12]. Consequently, developing green, sustainable cushioning materials has become a major research focus. For example, starch-based foam cushions are produced by foaming and molding starch into renewable, biodegradable packaging materials [7,13]. Plant-fiber bio-foams are made by mechanically entraining air into a fiber suspension to form a stable foam network, followed by high-temperature drying to lock in a porous, lightweight, low-density structure [14]. However, their production is complex and expensive.

Mycelium-based composite materials represent a novel, eco-friendly solution that is typically formed by combining fungal mycelia with a lignocellulosic matrix. During colonization, the mycelium continuously grows both into and around the substrate, binding it together into an integrated organic structure [15]. By employing molds of various shapes, it is possible to fabricate mycelium-based composites in configurations that meet specific design requirements. Upon drying, the material has a foam structure [16]. In early work, Arifin and Yusuf [17] produced mycelium composites on rice hulls and wheat bran, demonstrating the potential of fungal mycelium as a substitute binder for foam production. Fungal mycelium serves as a unique, natural binder that both grows on lignocellulosic substrates and integrates within the composite matrix [18]. Furthermore, the cultivation process requires minimal energy input and produces no by-products, enabling low-cost manufacturing [19]. Several studies and industry reports have highlighted the significant cost benefits of mycelium composites. They can reduce costs by more than 65% compared with paper-based materials and by more than 90% compared with gypsum-based, polymer, or wood–PHA composites [20]. Enarevba and Haapala [21] performed a life-cycle assessment (LCA) of packaging inserts made from EPS and mycelium. These results show that mycelium-based materials have a lower environmental impact than EPS does. Owing to their lightweight, biodegradable, and renewable nature, mycelium composites have attracted widespread interest and hold promise as alternatives to conventional synthetic and other non-renewable materials [22,23]. Lignocellulose, the structural component of both woody and nonwoody fibers, primarily comprises cellulose, hemicellulose, and lignin, serving as a crucial source of renewable organic matter [24]. However, the content and structure of cellulose, hemicellulose, and lignin vary according to the type of vegetation from which the lignocellulose is derived [25]. In particular, lignin, which is interspersed between cellulose and hemicellulose, enhances the hardness and mechanical strength of plant cell walls [26]. Cellulose, hemicellulose, and pectin are the key carbohydrates in the substrate, and they are partially consumed to supply nutrients for mycelial growth [27]. Mycelium-based composites are not only fully biodegradable but also enable the recycling and reuse of agricultural and forestry residues. Currently, an increasing volume of lignocellulosic waste generated by the agriculture and wood-processing industries is being utilized to produce mycelium-based composites. For example, sawdust, wheat straw, corn stalks, sugarcane bagasse, rice straw, cottonseed shells, and coconut husk fibers are among the recycled materials used [28,29,30]. Composites produced from these lignocellulosic materials have demonstrated favorable physical and mechanical properties; notably, those made with sugarcane bagasse as the base material exhibit mechanical performance comparable to that of expanded polystyrene (EPS) packaging materials [15]. Therefore, investigating the use of agricultural residues as substrates for producing mycelium-based composites could pave the way for the high-value utilization of such wastes, thereby increasing overall resource efficiency.

The performance of mycelium-based composites varies significantly depending on the substrate type, fungal species, and cultivation conditions. Preliminary experiments have been conducted to assess the performance of these materials. For example, Appels et al. [31] compared the cultivation of *Pleurotus ostreatus* and Trametes versicolor on three substrates, namely, beech sawdust, rapeseed straw, and cotton, and reported that both the fungal species and substrate type largely affect the stiffness and water resistance of the composites. Kuribayashi et al. [32] reported that fungal species dictate mycelial properties and that dense, continuous aerial hyphae enhance the flexibility and shape retention of mycelium-based composites. Similarly, Bruscato et al. [33] reported a correlation between the characteristics of fungal mycelia and compressive strength; leathery mycelia confer greater compressive strength than fragile hyphae, and composites grown on wood chips presented greater compressive strength than expanded polystyrene (EPS). Therefore, selecting strains with rapid growth, strong contamination resistance, storage stability, and environmental adaptability is essential to ensure the mechanical strength of composites. Strains such as *Trametes versicolor*, *Pleurotus ostreatus*, *Lentinula edodes*, and *Ganoderma lucidum* have been widely employed in numerous studies because of their superior performance [34,35,36]. Notably, substrate fiber dimensions, which are a critical processing parameter, play a key role in determining composite performance. Elsacker et al. [35] produced fibers of various sizes using techniques such as loosening, chopping, dust removal, pre-compression, and fiber bundling and observed that samples with smaller fiber sizes exhibited higher compressive moduli, while those containing short fibers had more cohesive and smoother outer layers. Furthermore, regarding thermal insulation, Schritt et al. [30] investigated the feasibility of producing mycelium-based insulation composites using a mushroom substrate and sawdust. Their results indicated that the material’s thermal conductivity ranged from 0.045 to 0.077 W/(m·K), demonstrating excellent insulating properties. Consequently, the development of packaging products based on mycelium-based composites holds considerable promise.

In this study, *Salix psammophila* and peanut straw were selected as substrates for fungal cultivation. *Salix psammophila* is a psammophytic shrub that grows rapidly, develops an extensive interwoven root system and dense branches, and is highly effective for windbreak and sand stabilization. In the Ordos region of Inner Mongolia alone, periodic mowing for rejuvenation yields approximately 400,000 tons of *Salix psammophila* biomass each year [37]. Peanut is an important oilseed crop and one of China’s key economic commodities. In China, peanut cultivation covers approximately 20% of the global planted area, with annual yields exceeding 13 million tons or approximately 40% of the world total, making China the largest peanut producer worldwide [38]. The efficient use of these resources can guide the high-value valorization of agricultural residues and enhance overall resource efficiency.

Mycelium-based composites show considerable potential for use in cushioning packaging. However, prior to this study, there have been no reports on the use of sand willow or peanut straw as substrates for fabricating such materials. This study aims to develop biodegradable and sustainable cushioning materials as environmentally friendly alternatives to conventional plastic foams. This research focuses on cultivating two representative fungal species, *Pleurotus ostreatus* and *Ganoderma lucidum*, on two distinct lignocellulosic substrates to fabricate *Pleurotus ostreatus* mycelium–*Salix psammophila* cushion material (POSM) and *Ganoderma lucidum* mycelium–peanut straw cushion material (GLPM). This study evaluated the effects of the cultivation time and substrate length on the physical and mechanical properties of mycelium-based composites, including compressive strength, resilience, water contact angle, and thermal insulation performance. It also compares the performance of materials derived from different lignocellulosic sources. In addition, thermogravimetric analysis (TGA), scanning electron microscopy (SEM), and Fourier transform infrared spectroscopy (FTIR) were conducted to investigate the thermal stability, microstructure, and chemical composition of the materials, providing further insight into their potential as cushioning packaging materials. These findings offer valuable insights into the viability of mycelium-based materials as sustainable substitutes for traditional plastic foams in cushioning applications.

## 2. Materials and Methods

### 2.1. Materials

The *Pleurotus ostreatus* and *Ganoderma lucidum* strains were purchased from Junzhiyuan Co., Ltd., Guang’an, China. The *Pleurotus ostreatus* strain was obtained by culturing on solid media composed of 63% cottonseed hulls, 30% corn cob powder, 5% wheat bran, 1% gypsum, and 1% sucrose for 20 days. The *Ganoderma lucidum* strain was obtained by culturing on a solid medium composed of 75% oak sawdust, 23% wheat bran, 1% sucrose, and 1% gypsum for 20 days. Peanut straw was collected from Ulanhot in Hinggan League, Inner Mongolia Autonomous Region. *Salix psammophila* was harvested from the Maowusu Desert, Ordos city, Inner Mongolia. Dried cattle dung was sourced from herder households in Jinhe Town, Hohhot, Inner Mongolia, where cattle are primarily fed on forage. Gypsum powder (purity ≥ 90%, particle size < 150 µm) was purchased from Chenglin Fungal Industry Co., Ltd., Jingshan, China. Food-grade corn starch was supplied by Bolin Biotechnology Co., Ltd., Xi’an, China. Wheat bran was procured from Linjing Trading Co., Ltd., Jinan, China.

### 2.2. Preparation of Mycelium-Based Cushioning Materials

**Figure 1:**
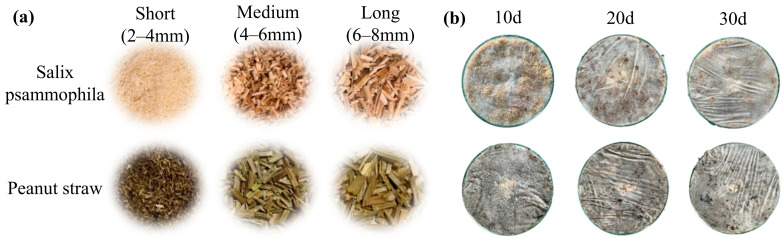
(**a**) *Salix psammophila* and peanut straw with different substrate lengths; (**b**) mycelial growth at different cultivation times.

Mycelium-based cushioning materials are produced from fungi and agricultural residues. The different substrate lengths of *Salix psammophila* and peanut straw (2–4 mm, 4–6 mm, and 6–8 mm) are shown in Figure 1. All tools were sterilized prior to cultivation. *Salix psammophila* and peanut straw substrates were prepared on the basis of the strain–substrate adaptation results provided in the Supplementary Materials (Figures S1–S5). A mixture of 70% *Salix psammophila*, 20% cattle manure, 2% gypsum, and 8% starch was prepared. A second mixture containing 70% peanut straw, 20% wheat bran, 2% gypsum, and 8% starch was prepared. For each 100 g of substrate, 110 mL of water was added. After thorough mixing, the substrates were placed into polypropylene bags and sterilized at 121 °C for 50 min in a steam autoclave (LS11-12B, Sunne, Shanghai, China). After cooling, the *Pleurotus ostreatus* and *Ganoderma lucidum* strains were inoculated into the *Salix psammophila* and peanut straw substrates, respectively, at a 20% mass fraction. The inoculated substrates were then placed into molds (100 mm × 100 mm × 50 mm) and tightly covered with plastic wrap, with a series of small perforations made on the wrap via a fine needle. The molds were then placed in a constant temperature and humidity chamber (HWS-80B, Mingtu Machinery Equipment, Changge, China) maintained at 25 °C with a relative humidity of 60%. Under these conditions, the samples were incubated for 10, 20, or 30 days. After cultivation, the plastic wrap was removed, and the materials were demolded. During cultivation, the cultivation environment was disinfected regularly with 75% ethanol to prevent contamination of the materials. Finally, the mycelium-based cushioning materials were dried in an oven (101-3B, Changge, China) at 90 °C for 7 h.

### 2.3. Morphological Characterization of Mycelium-Based Cushioning Materials

Scanning electron microscopy (S-3400 N, Tokyo, Japan) was employed to characterize the pure mycelial morphology as well as the internal microstructure of the mycelium-based cushioning materials. Prior to imaging, the sample surfaces were gold-sputtered under vacuum to enhance conductivity, and the observations were made at an accelerating voltage of 20 kV.

### 2.4. Infrared Spectroscopy Testing

*Salix psammophila*, peanut straw, and the two mycelium-based cushioning material samples were ground into fine powder. A Fourier transform infrared (FTIR) spectrometer (ALPHA II, BRUKER, Berlin, Germany) was used to analyze molecular structure variations and assess the functional groups in the powdered samples. Spectra were recorded over a wavenumber range of 4000–400 cm^−1^ with each sample scanned 64 times at a resolution of 4 cm^−1^. Three replicates of each sample type were measured to ensure reproducibility.

### 2.5. Thermal Stability Testing

The two mycelium-based cushion material samples were ground into a fine powder. Thermal gravimetric analysis was performed via a thermogravimetric analyzer (TGA-4000, Perkin Elmer, Waltham, MA, USA). For each test, approximately 5 mg of powder from each sample was placed in an alumina crucible, with nitrogen serving as the carrier gas. The heating rate was set to 10 °C/min, and the gas flow rate was 50 mL/min, with the samples heated from room temperature to 700 °C.

### 2.6. Density

The density was determined following GB/T 8168-2008 “Testing Method of Static Compression for Packaging Cushioning Materials” [39]. The sample mass was recorded with an electronic balance, and the thickness was measured via a Vernier caliper. Before measuring the thickness, a rigid plate was placed atop each sample to apply a compressive load of 0.20 kPa ± 0.02 kPa. After 30 s under loading, the thickness of the four corners was measured. Four replicate tests were conducted for each type, and the mean value was calculated. The density was calculated via Equation (1).

(1) $$\rho =\frac{m}{ST}$$

where ρ is the density (g/cm^3^), *m* is the sample mass (g), *S* is the base area of the sample (cm^2^), and *T* is the sample thickness (cm).

### 2.7. Compressive Strength Testing

Static compression tests were carried out in accordance with GB/T 8168-2008 “Testing Method of Static Compression for Packaging Cushioning Materials”. Before each test, the specimen thickness was measured to determine the initial thickness (T). Compression was performed on a universal testing machine (FFiber-WL8007, FFIBER, Guangdong, China) with a 2 kN capacity using samples approximately 90 mm × 90 mm × 40 mm in size. A platen applied load was applied at 12 mm/min along the thickness direction until the sample deformed by 50%. Load–displacement data were converted to stress (*σ*)–strain (*ε*) curves via Equations (2) and (3). Four replicate tests were conducted for each type, and the mean value was calculated.

(2) $$\sigma =\frac{F}{A}$$

where *σ* is the compressive stress (MPa), *F* is the applied load (N), and *A* is the base area of the sample (mm^2^).

(3) $$\varepsilon =\frac{T-T_{j}}{T}$$

where *ε* is the compressive strain (%), *T* is the original specimen thickness (mm), and *T_j_* is the specimen thickness at each compression step (mm).

### 2.8. Elastic Spring Back Rate Testing

In accordance with GB/T 8168-2008 “Testing Method of Static Compression for Packaging Cushioning Materials”, testing was halted at 50% strain. After unloading and a 3 min hold, the rebound thickness was measured with a digital Vernier caliper (JS20F300, SYNTEK, Jingzhou, China). Four replicate tests were conducted for each type, and the mean value was calculated. The elastic spring back rate was calculated via Equation (4).

(4) $$w=\frac{T_{j}-\left(\frac{T}{2}\right)}{\frac{T}{2}}$$

where *w* is the elastic spring back rate (%), *T* is the initial thickness before compression (mm), and *T_j_* is the thickness after recovery (mm).

### 2.9. Water Contact Angle Measurement

A contact angle meter (SDC-100, Xiwak, Dongguan, China) was used to perform water contact angle measurements on the surfaces of the mycelium-based cushioning materials. Following the GB/T 30693-2014 “Measurement of Water Contact Angles of Plastic Films” standard [40], specimens with smooth surfaces were selected, and a droplet was manually and slowly deposited onto each surface to ensure a uniform droplet size and shape. During measurement, approximately 20 μL of water was dispensed onto randomly selected areas of the sample surfaces. Four samples of each type were chosen, with each sample undergoing four repeated tests; the average value was then calculated.

### 2.10. Thermal Conductivity Measurement

The thermal conductivity of the samples was measured via a thermal conductivity analyzer (Mobile M10 typ1, ai-Phase, Tokyo, Japan). The test was conducted at an average temperature of 25 °C. A periodic heating method, based on the propagation and attenuation of thermal waves, was employed to determine the thermal conductivity by analyzing the heat wave diffusion behavior within the material. Four samples of each type were chosen, with each sample undergoing ten repeated tests; the average value was then calculated.

### 2.11. Statistical Analysis

The data were processed in Microsoft Excel, and one-way ANOVA was conducted in SPSS 16.0 to evaluate the experimental results. *p* ≤ 0.05 was considered statistically significant.

## 3. Results and Discussion

### 3.1. Morphological Analysis of Mycelium-Based Cushioning Materials

**Figure 2:**
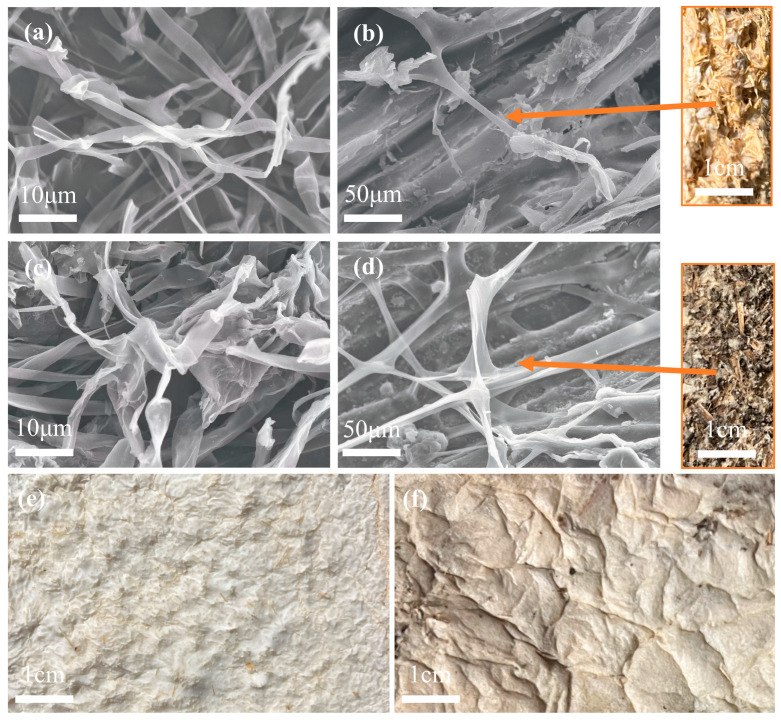
Images of mycelium-based cushioning materials derived from different lignocellulosic substrates: (**a**,**c**) show the microscopic morphology of *Pleurotus ostreatus* and *Ganoderma lucidum* mycelia, respectively (3000×); (**b**,**d**) display the cross-sectional morphology and internal microstructure of POSM and GLPM, respectively (1000×); (**e**,**f**) present the surface morphology of POSM and GLPM, respectively (1×).

Figure 2 presents scanning electron micrographs of the two mycelium-based cushioning materials after 30 days of cultivation. Figure 2a,c show the microscopic morphology of the *Pleurotus ostreatus* and *Ganoderma lucidum* mycelia, respectively. Both materials exhibit a flattened cavity structure; however, the *Ganoderma lucidum* mycelium has a relatively large diameter. Fungi facilitate mycelial growth by degrading the substrate through the secretion of lignin peroxidase, cellulase, and laccase [41]. During growth, the mycelium develops branched structures that intertwine and aggregate, ultimately forming a three-dimensional network. This densely interwoven mycelial layer is responsible for the excellent water resistance observed on the surface of the cushioning materials.

Figure 2b,d present the internal morphology of POSM and GLPM. In both materials, hyphae adhere closely to the substrate and interweave to bind the originally loose particles into a cohesive matrix, yielding strong mechanical properties. Cross-sectional images revealed internal voids in both cushioning materials. Compared with POSM, GLPM results in a greater mycelial density, a difference that may directly affect the bulk density, compressive strength, and elastic spring. These properties are governed by the structure of the mycelial binding network and hyphal density [34]. Figure 2e,f illustrate the surface morphology of POSM and GLPM. Both cushioning materials form a dense mycelial film. Surface colonization is visibly denser than in the interior, likely because higher oxygen availability at the surface promotes hyphal growth. However, limited airflow into the core restricts internal colonization [42]. Additionally, the surface films differ in color: POSM appears white, whereas GLPM is light brown. This variation may result from polysaccharide caramelization or the thermal degradation of organic compounds during drying [43].

### 3.2. Sample Functional Group Analysis

**Figure 3:**
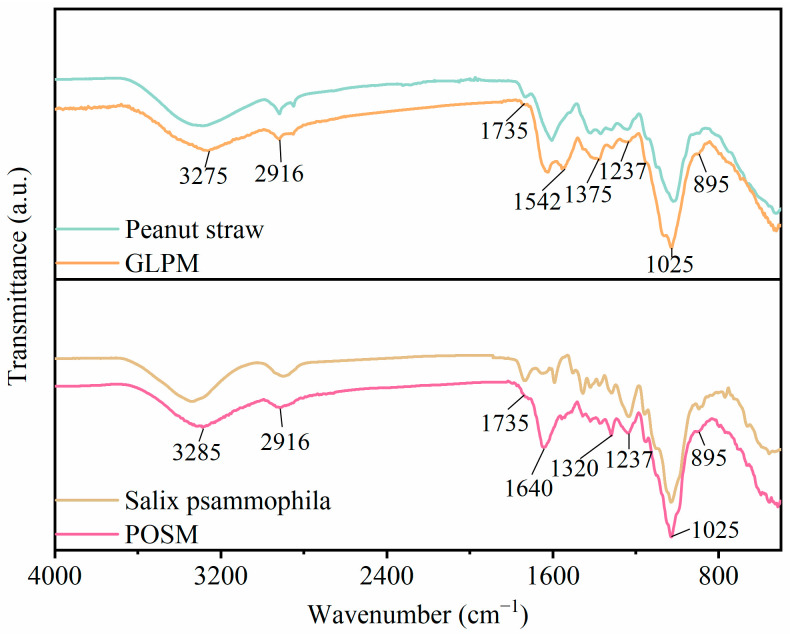
FTIR spectra of *Salix psammophila*, peanut straw, and mycelium-based cushioning materials.

Figure 3 presents the FTIR spectra of *Salix psammophila*, peanut straw, POSM, and GLPM over the wavenumber range of 4000–400 cm^−1^. After fungal stabilization, significant differences in spectral intensities were observed among *Salix psammophila*, peanut straw, and the two mycelium-based cushioning materials. Among the main characteristic peaks in *Salix psammophila* and peanut straw, the absorption peak at 3200–3400 cm^−1^ corresponds to the stretching vibration of hydroxyl (–OH) groups, which primarily originate from the cellulose and hemicellulose in these substrates. The absorption peak between 3000 and 2800 cm^−1^ corresponds to the stretching vibrations of C–H bonds in lipid compounds. The absorption peak at 1735 cm^−1^ is attributed to the stretching vibration of nonconjugated C=O bonds in the acetyl groups of hemicellulose. The absorption peak in the 1500–1200 cm^−1^ range represents the stretching vibrations of the aromatic ring skeleton in lignin. Gelbrich et al. [44] demonstrated a linear correlation between lignin content and peak intensity in lignocellulosic materials. The absorption peak between 1200 and 900 cm^−1^ corresponds to the stretching vibrations of C–O, C–C, and C–O–C bonds, which are associated with the sugar ring structures in the cellulose and hemicellulose of the substrate [15]. The characteristic peak at 895 cm^−1^ is typically attributed to the vibrational absorption of β-glycosidic bonds in cellulose [45].

The FTIR spectra of the mycelium-based cushioning materials are influenced by the biomolecules present in both the mycelium and the substrate. The characteristic peaks of the fungal mycelium mainly include those associated with lipids (3000–2800 cm^−1^), proteins (Amide I: 1700–1600 cm^−1^; Amide II and III: 1575–1300 cm^−1^), chitin (1318–1415 cm^−1^), nucleic acids (1255–1245 cm^−1^), and polysaccharides (1200–900 cm^−1^) [46,47]. The primary biomolecular peaks are observable in the mycelium-based cushioning materials, and the presence of chitin contributes to enhanced mechanical properties [48]. For example, at 1640 cm^−1^ and 1542 cm^−1^, both materials exhibit enhanced absorption peaks, primarily due to the stretching vibrations of C=O or C=C, N–H, and C–N groups in amide I and amide II. Notably, this enhancement is more pronounced in POSM. The absorption peaks at 1320 cm^−1^ and 1375 cm^−1^ correspond to the C–H stretching vibrations of chitin in POSM and GLPM, respectively. At 1025 cm^−1^, both cushioning materials presented increased absorption peaks, likely due to the polysaccharides present in the fungal mycelium. Haneef et al. [47] reported that these polysaccharides confer rigidity to the mycelial film. Additionally, compared with the absorption peaks of *Salix psammophila* and peanut straw, those of the mycelium-based cushioning materials exhibited reduced absorption in the 3200–3400 cm^−1^ range, possibly due to the reduction in the number of active hydroxyl groups by laccase during fungal growth. Notably, the signal in the 3200–3400 cm^−1^ range was significantly lower in POSM than in *Salix psammophila*, indicating that the growth of *Pleurotus ostreatus* mycelia consumes greater amounts of cellulose and hemicellulose from the substrate. Furthermore, the absorption peaks at 2916, 1735, 1500–1200, and 895 cm^−1^ all exhibit varying degrees of intensity reduction, which is consistent with the findings of Peng et al. [15], and is attributable to the degradation of lignin, hemicellulose, and cellulose in the substrate. Specifically, lignin, a complex aromatic polymer, undergoes partial depolymerization during fungal colonization, resulting in a decrease in aromatic and phenolic moieties. Hemicellulose comprises heterogeneous polysaccharides (including xylose and mannose units) that are hydrolyzed by hemicellulases into smaller sugars, whereas cellulose, a crystalline polymer, is enzymatically cleaved by cellulases into glucose and cellobiose [49].

### 3.3. Thermal Performance Analysis

**Figure 4:**
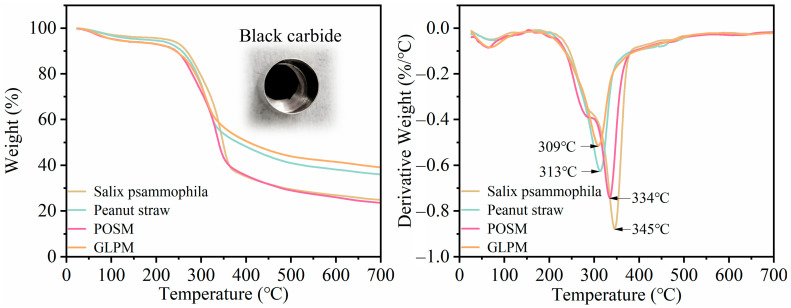
TG and DTG curves of *Salix psammophila*, peanut straw, and mycelium-based cushioning materials.

Thermogravimetric analysis (TGA) was conducted on the samples to evaluate the thermal stability of the materials. The thermogravimetric (TG) and derivative thermogravimetric (DTG) curves of *Salix psammophila*, peanut straw, POSM, and GLPM are presented in Figure 4. Supplementary Figures S6 and S7 present TG and DTG curves plotted against heating duration, illustrating the time required to reach each temperature. The mycelium-based cushioning materials exhibited thermal degradation behavior similar to that of their lignocellulosic substrates, which is consistent with the degradation temperature ranges reported in previous studies [50]. The mass loss process can be broadly divided into three stages. The first stage occurs between 25 °C and 180 °C, during which approximately 6–8% mass loss is observed due to the evaporation of water and volatile compounds [51].

In the second stage, the temperature ranges associated with thermal degradation differ among the materials. For *Salix psammophila* and POSM, mass loss occurred between 180 °C and 370 °C, whereas for peanut straw and GLPM, it occurred between 180 °C and 340 °C, with all samples reaching their maximum mass loss during this stage. The DTG curves indicate that the degradation rates of the two mycelium-based cushioning materials are lower than those of *Salix psammophila* and peanut straw. Notably, GLPM results in a lower mass loss than does peanut straw. During this stage, hemicellulose degrades rapidly between 220 and 315 °C and cellulose between 300 and 400 °C, whereas lignin degrades slowly over a broad temperature range (150–900 °C) [50,52]. Additionally, components in the mycelium, such as chitin, amino acids, carbohydrates, and glucans, begin to degrade at approximately 270 °C [53]. The mycelium-based cushioning materials produced from *Salix psammophila* and peanut straw exhibit distinct thermal degradation behaviors. The TG curves revealed that *Salix psammophila* and POSM experienced approximately 55% mass loss, whereas peanut straw and GLPM lost approximately 35% of their mass, which may be attributed to differences in the chemical composition of the substrates. *Salix psammophila* is typically composed of 52.63% cellulose, 22.3% hemicellulose, and 19.1% lignin [54]. In contrast, peanut straw contains 42.9% cellulose, 14.55% hemicellulose, and 17.49% lignin [55].

In the third stage, degradation in *Salix psammophila* and POSM occurred above 370 °C, whereas degradation in peanut straw and GLPM occurred above 340 °C. As the temperature increases, the residual mass levels off, and the lignin is nearly completely degraded. The final residual masses were 24% and 39%, respectively, and the remaining residue appeared as black carbide. Notably, the mycelium-based cushioning materials prepared in this study exhibit thermal degradation behavior comparable to that of most synthetic foams, which typically degrade in the range of 250 °C–475 °C [34]. However, compared with expanded polystyrene (EPS), which has a maximum degradation rate at approximately 440 °C according to Bruscato et al. [33], EPS has greater thermal stability.

### 3.4. Mechanical Performance Analysis

#### 3.4.1. Effects of Cultivation Time on the Performance of Mycelium-Based Cushioning Materials

**Figure 5:**
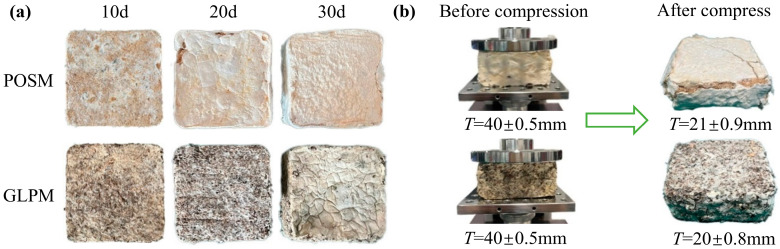
Mycelium-based cushioning materials at various cultivation times and before and after compression: (**a**) morphological characterization at different cultivation times; (**b**) comparison of the materials before and after compression after 30 days of mycelial cultivation.

Figure 5a shows the cushioning materials after 10, 20, and 30 days of cultivation. Visually, both materials exhibited progressively denser mycelial growth with increasing cultivation time. By day 30, the surface coverage of POSM was notably greater than that of GLPM. As shown in Table 1, the densities of both materials showed minimal variation with increasing cultivation time, and their values remained comparable. Peng et al. [15] reported that the density trend of mycelium composites generally aligns with that of the substrate prior to inoculation. In addition, Schritt et al. [30] reported that fungal colonization leads to substrate biomass degradation, and vigorous mycelial growth may contribute to a reduction in composite density. Figure 5b shows a comparison of the materials before and after compression following 30 days of cultivation. No lubricant was applied between the sample and the compression plates during the test. After compression testing, both materials exhibited reduced thicknesses and varying degrees of structural damage.

In this study, the compressive strength of mycelium-based cushioning materials produced from different lignocellulosic substrates varied, which is consistent with previous findings [34,35]. Specifically, POSM exhibited greater strength than GLPM. The compressive strength of these materials is highly dependent on both the substrate and the fungal mycelium, which also influences their morphology. Notably, the chitin present in the fungal cell wall enhances the mechanical strength of cushioning materials by aggregating primary fibers that support the structure and reduce crack formation under compression [48]. Proteins and lipids may act as plasticizers, whereas polysaccharides impart rigidity to the mycelial protective layer [36].

**Figure 6:**
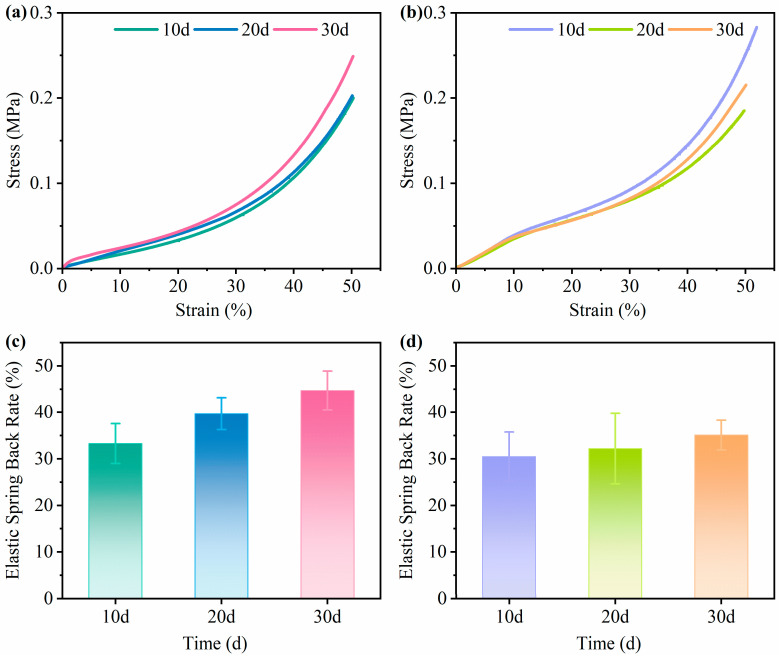
Stress–strain curves and elastic spring back rates of mycelium-based cushioning materials at different cultivation times: (**a**,**c**) for POSM; (**b**,**d**) for GLPM (*p* < 0.05).

Figure 6a,b show the stress–strain curves of the mycelium-based cushioning materials at various cultivation times, whereas Figure 6c,d present their corresponding elastic spring back rates. As the strain increases, the compressive stress in both cushioning materials progressively increases. The compressive strength of the mycelium-based materials increased linearly at low strain levels and increased more sharply as the external force increased. The compressive stress responses of the two materials varied depending on the cultivation duration. Table 1 shows the compressive strength of the mycelium-based materials at different cultivation periods. Under the same strain conditions, the compressive stress of POSM increased with an increasing cultivation time, with strength values ranging from 0.19 to 0.25 MPa (an increase of 31.5%). In contrast, GLPM showed a decreasing–increasing trend, with the highest compressive strength of 0.27 MPa observed at 10 days of cultivation. With respect to elastic spring back performance, both materials showed improved resilience with longer cultivation, increasing from 33.3% to 44.7% (a 34.2% increase) for POSM and from 30.5% to 35.1% (a 15% increase) for GLPM.

At 10 days of cultivation, GLPM exhibited greater compressive stress. As shown in Figure 1b, the mycelia of *Ganoderma lucidum* grown on the peanut straw substrate appeared more abundant than the mycelia of *Pleurotus ostreatus* on the sand willow substrate. This may be attributed to the lower lignin content in peanut straw, which allows for more rapid lignin degradation and generates a higher concentration of free sugars, thereby promoting faster fungal mycelial growth [56]. In contrast, at 10 days, POSM has a lower mycelial density both internally and externally, with a looser structure that is more prone to collapse under compression. After 20 days of cultivation, further mycelial growth was observed. The compressive stress of POSM gradually increased with prolonged cultivation, whereas that of GLPM decreased significantly, possibly due to the additional degradation of cellulose, hemicellulose, and lignin in the substrate, which undermined the integrity of the fibers [51]. Although the compressive stresses of the two materials were comparable, both demonstrated a marked improvement in elastic spring back performance, reaching 39.7% and 32.2%, respectively. This increase can be attributed to the development of a denser mycelial network. At 30 days of cultivation, both types of cushioning materials presented increased compressive stress compared with those incubated for 20 days. Additionally, their elastic spring back performance peaked at 44.7% for POSM and 35.1% for GLPM. As shown in Figure 5a, both materials developed a thick mycelial layer that encapsulated the substrate, giving the surface a flexible, leather-like appearance, which is beneficial for enhancing mechanical performance [57]. However, prolonged cultivation led to further substrate degradation by the fungus, resulting in a lower compressive stress for GLPM than for the 10-day sample [58]. These findings are consistent with those of previous studies, which suggest that the mechanical strength of mycelium-based composites varies depending on the fungal species and the type of lignocellulosic substrate used. Aiduang et al. [34] reported that mycelium composites made from sawdust presented greater compressive strength than those made from corn husks or rice straw across different fungal species. Similarly, Ghazvinian et al. [59] reported that sawdust-based mycelium composites had higher compressive strength than those made with straw. Moreover, differences in the mechanical characteristics of fungal mycelia may be attributed to variations in their chemical composition and microstructure. The proportions of chitin, proteins, lipids, and polysaccharides in the mycelium influence its morphology and are closely related to the mechanical strength of the composite [58]. Haneef et al. [47] reported that materials derived from *Pleurotus ostreatus* mycelia were stiffer and exhibited lower elongation at break than those derived from *Ganoderma lucidum*. In summary, both cushioning materials demonstrated favorable mechanical properties after 30 days of cultivation.

#### 3.4.2. Effects of Substrate Length on the Performance of Mycelium-Based Cushioning Materials

In this study, the mycelium-based cushioning materials demonstrated robust physico-mechanical properties after 30 days of cultivation. Consequently, all samples with different substrate lengths were cultivated for 30 days. Appels et al. [31] reported that the density of mycelium-based materials is influenced by both the fungal species and the substrate composition. As shown in Table 2, in our experiments, the densities ranged from 0.13 to 0.16 g/cm^3^ and decreased as the substrate length increased. These values lie within the previously reported density range for mycelium composites (25–954 kg/m^3^) [60]. However, compared with expanded polystyrene (11–50 kg/m^3^), mycelium-based cushioning materials presented higher densities. Therefore, controlling the density of mycelium-based cushioning materials remains a considerable challenge [57].

**Figure 7:**
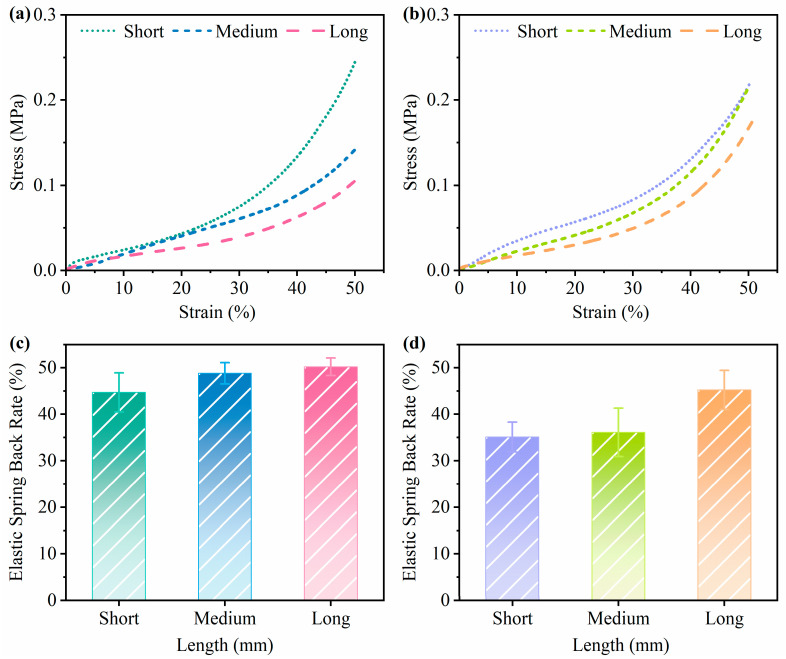
Stress–strain curves and elastic spring back rates of mycelium-based cushioning materials with different substrate lengths: (**a**,**c**) for POSM, (**b**,**d**) for GLPM (*p* < 0.05).

Figure 7a,b show the stress–strain curves of mycelium-based cushioning materials with different substrate lengths after 30 days of cultivation. Figure 7c,d display the elastic spring back rates of these materials after 30 days of cultivation. Table 2 shows the compressive strength of mycelium-based cushioning materials with different substrate lengths. Under identical strain conditions, the compressive stress decreased with increasing substrate length in both materials, from 0.25 MPa to 0.10 MPa (a 60% reduction) and from 0.21 MPa to 0.17 MPa (a 19.04% reduction), respectively. This may be attributed to the ability of short *Salix psammophila* and peanut straw particles to pack more densely and support each other more effectively. In contrast, as the substrate length increases, the material is more prone to bending, misalignment, and sliding under pressure, which reduces structural stability and makes the substrate more susceptible to deformation, resulting in lower compressive stress. Furthermore, Soh et al. [61] reported that smaller substrate particles may promote better mycelial growth and enhance compressive strength. With respect to elastic spring back performance, cushioning materials prepared from long *Salix psammophila* and long peanut straw exhibit superior elastic spring back characteristics, with elastic spring back rates of 50.2% and 45.2%, respectively. This may be due to the greater elastic deformation capacity of the substrate under pressure, which allows it to better recover its original form once the pressure is released. Additionally, the proteins and lipids in fungal mycelia contribute to improved elastic recovery, enhancing the ability of the material to regain its form [47]. In this study, the compressive strength of mycelium-based cushioning materials with varying substrate lengths ranged from 0.1 to 0.25 MPa, which aligns with the values reported by Zhang et al. [58], ranging from 0.16 to 0.26 MPa. Moreover, the obtained compressive strength values fall within the range of expanded polystyrene (EPS), which ranges from 0.03 to 0.69 MPa [60]. These results suggest that the mycelium-based cushioning materials developed in this study have potential for replacing conventional foam packaging materials.

### 3.5. Water Contact Angle and Thermal Conductivity Analysis

**Figure 8:**
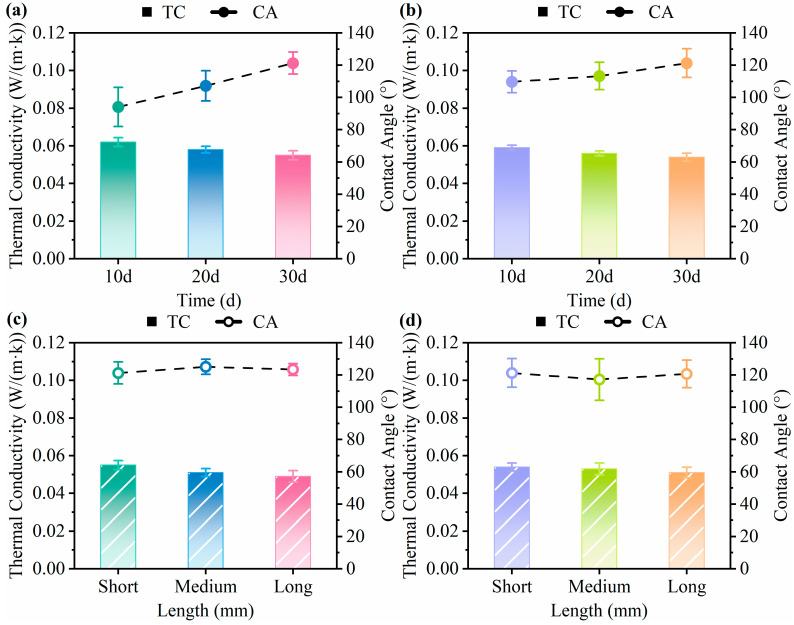
Thermal conductivity and contact angle of mycelium-based cushioning materials with different cultivation times and substrate lengths: (**a**,**c**) for POSM, (**b**,**d**) for GLPM (*p* < 0.05).

**Figure 9:**
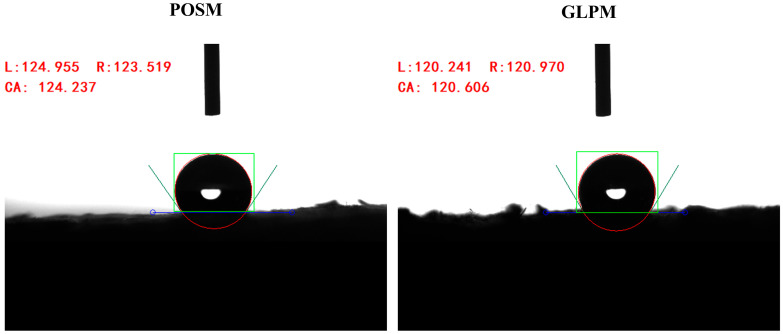
Static water contact angles of the mycelium-based cushioning materials.

Figure 8a,b show the water contact angles and thermal conductivity of the mycelium-based cushioning materials at various cultivation times. Figure 8c,d show the water contact angles and thermal conductivity of the mycelium-based cushioning materials prepared with different substrate lengths after 30 days of cultivation. The water contact angle is the angle formed when a water droplet contacts a material surface. When the contact angle exceeds 90°, the surface is hydrophobic, causing the droplet to retain a nearly spherical shape [62]. Lignocellulose is typically hydrophilic due to the abundance of accessible hydroxyl groups in its cellulose constituents [29,63]. The density of the mycelial network and the presence of aerial hyphal biofilms increase surface hydrophobicity. Aerial hyphae contain hydrophobin proteins that impart hydrophobic characteristics to the surface [64,65]. Zhang et al. [58] reported that composites lacking a well-developed aerial hyphal layer exhibited poorer hydrophobicity. As shown in Figure 8, all the samples presented contact angles above 90°, indicating a degree of water resistance. Therefore, the formation of aerial hyphae on the lignocellulosic surface can significantly alter its wettability [66]. With prolonged cultivation, surface mycelia differentiated into dense, uniform biofilms, resulting in progressively higher contact angles for both materials. After 30 days of cultivation, both materials displayed contact angles exceeding 120°. Figure 9 shows representative static water contact angle images. These results align with the 123.17° reported by Zhang et al. [58] and exceed the 113.9° measured for polypropylene by Izan et al. [67], which may be advantageous for moisture-resistant applications. As the substrate length increased, the contact angle changed minimally, increasing from 121.5° to 123.4° (a 1.56% increase) and decreasing from 121.3° to 120.7° (a 0.49% decrease). This finding indicates that the substrate length has little effect on the hydrophobicity of the material surface, likely because larger particles do not significantly alter aerial hyphal development.

To assess the thermal insulation performance of the mycelium-based cushioning materials, we measured their thermal conductivities. The thermal conductivity indicates a material’s ability to conduct heat under steady-state conditions. Materials with values less than 0.07 W/(m·K) are generally classified as thermal insulators. The mycelium forms a complex network of intertwined, slender hyphae filled with fine pores, which effectively reduce heat flow [68]. With prolonged cultivation, the fungus progressively degrades the substrate, particularly the highly conductive lignin and hemicellulose [69]. Zhang et al. [58] reported that fungal activity also creates micro-pits in cell walls. These changes reduce the continuity of the internal solid matrix and consequently lower the material’s thermal conductivity. After 30 days of cultivation, both cushioning materials reached their minimum thermal conductivities, which were 0.055 and 0.054 W/(m·K), respectively. As the substrate length increased, the thermal conductivities of both materials decreased slightly to 0.049 and 0.051 W/(m·K). This likely reflects an increase in void space, which further restricts heat transfer through the solid phase. The measured values align with previously reported data (0.04–0.057 W/(m·K)) [35,58]. Moreover, these conductivities approach those of expanded polystyrene (0.03–0.04 W/(m·K)), indicating that mycelium-based cushioning materials offer comparable insulation performance [60,70].

## 4. Conclusions

In this study, *Salix psammophila* and peanut straw substrates were inoculated with *Pleurotus ostreatus* and *Ganoderma lucidum* to produce POSM and GLPM, respectively. These mycelium-based cushions offer a sustainable alternative to conventional foams, reducing the environmental impact and dependence on nonrenewable resources. We further investigated how the cultivation time and substrate length affect density, contact angle, thermal conductivity, thermal stability, and mechanical properties (compressive strength and elastic spring back rate). The key findings are as follows:

1. Mycelium-based cushioning material performance varies with the lignocellulose type and fungal species, with POSM outperforming GLPM overall.
2. Mycelium-based cushioning materials exhibit thermal degradation profiles similar to those of synthetic foams and relatively high thermal stability.
3. Longer cultivation improved the physical and mechanical properties, particularly surface hydrophobicity, as a result of the development of a mycelial biofilm. Overall, incubating the fungi for 30 days yielded superior performance. However, further cultivation could degrade the mechanical strength.
4. Increasing the substrate length improved most performance metrics of the mycelium-based cushioning materials. However, samples made with larger substrate particles exhibited lower compressive strengths.

Mycelium-based cushioning materials derived from agricultural residues are eco-friendly and biodegradable, holding promise as green packaging alternatives. Nonetheless, their low mechanical strength remains a significant barrier to their widespread adoption. Future work should evaluate additional fungal species to identify those offering optimal degradation and reinforcement, improving fungus–substrate compatibility. In parallel, incorporating reinforcing additives into the substrate may enhance the mechanical performance. Moreover, the long cultivation period reduces economic feasibility. Future efforts should explore exogenous nutrient supplementation to shorten the growth cycle and enable rapid, large-scale production. This study demonstrates the considerable potential of fungal biotechnology for manufacturing eco-friendly packaging materials.

## Figures and Tables

**Table 1:** Density and compressive strength of mycelium-based cushioning materials at different cultivation times (*p* < 0.05).

Time (d)	POSM		GLPM	
	Density (g/cm^3^)	Compression Strength (MPa)	Density (g/cm^3^)	Compression Strength (MPa)
10	0.151 ± 0.0057	0.19 ± 0.012	0.153 ± 0.0037	0.27 ± 0.012
20	0.157 ± 0.0047	0.20 ± 0.022	0.155 ± 0.0042	0.17 ± 0.022
30	0.164 ± 0.0067	0.25 ± 0.017	0.163 ± 0.0060	0.21 ± 0.030

**Table 2:** Density and compressive strength of mycelium-based cushioning materials with different substrate lengths (*p* < 0.05).

Length (mm)	POSM		GLPM	
	Density (g/cm^3^)	Compression Strength (MPa)	Density (g/cm^3^)	Compression Strength (MPa)
Short (2–4)	0.164 ± 0.0067	0.25 ± 0.017	0.163 ± 0.0060	0.21 ± 0.030
Medium (4–6)	0.152 ± 0.0047	0.14 ± 0.020	0.158 ± 0.0074	0.20 ± 0.023
Long (6–8)	0.132 ± 0.0058	0.10 ± 0.035	0.133 ± 0.0038	0.17 ± 0.014

## Data Availability

The original contributions presented in this study are included in the article/Supplementary Materials. Further inquiries can be directed to the corresponding author(s).

## Supplementary Materials

The following supporting information can be downloaded at: https://www.mdpi.com/article/10.3390/biomimetics10060371/s1, Figure S1: The growth of *Ganoderma lucidum* mycelium on different carbon sources; Figure S2: The growth of *Lentinula edodes* mycelium on different carbon sources; Figure S3: The growth of *Pleurotus ostreatus* mycelium on different carbon sources; Figure S4: The growth of *Ganoderma lucidum* mycelium on different nitrogen sources; Figure S5: The growth of *Pleurotus ostreatus* mycelium on different nitrogen sources; Figure S6: The temperature–time profile during thermogravimetric analysis (TGA); Figure S7: TG and DTG curves as a function of temperature.
