# Supplementary material for: The Effects of the Substrate Length and Cultivation Time on the Physical and Mechanical Properties of Mycelium-Based Cushioning Materials from Salix psammophila and Peanut Straw
Source: Biomimetics (Basel). 2025 Jun 5;10(6):371. doi: 10.3390/biomimetics10060371 (PMC12191277; doi:10.3390/biomimetics10060371)
Supplement: Supplementary file 1 [file biomimetics-10-00371-s001.zip › biomimetics-3626902-supplementary.pdf]

The Effects of the Substrate Length and Cultivation Time on the Physical and Mechanical Properties of Mycelium-Based Cushioning Materials from *Salix psammophila* and Peanut Straw

Xiaowen Song [1,2], Shuoye Chen [1,2], Jianxin Wu [1,2,*], Ziyi Cai [3,*], Yanfeng Zhang [1,2], Risu Na [1,2], He Lv [1,2], Cong He [1,2], Tingting Wu [3] and Xiulun Wang [3]

1 College of Mechanical Engineering, Inner Mongolia University of Technology, Hohhot 010051, China; sxw_istgut@imut.edu.cn (X.S.); 20231800013@imut.edu.cn (S.C.); zhyf_2023@imut.edu.cn (Y.Z.); nrs3000@imut.cn (R.N.); lvhe@imut.edu.cn (H.L.); 20211100020@imut.edu.cn (C.H.)
2 Inner Mongolia Key Laboratory of Robotics and Intelligent Equipment Technology, Inner Mongolia University of Technology, Hohhot 010051, China
3 Graduate School of Bioresources, Mie University, 1577 Kurimamachiya-cho, Tsu 5148507, Japan; wu@bio.mie-u.ac.jp (T.W.); wang@bio.mie-u.ac.jp (X.W.)
* Correspondence: wujx@imut.edu.cn (J.W.); caiziyi@bio.mie-u.ac.jp (Z.C.)

Supplement

1 Carbon source and strain adaptation

Based on a standard solid culture medium with a mass ratio of 70:20:2:8:110 for carbon source, bran, gypsum, starch, and water, the components were thoroughly mixed and packed into polypropylene bags for sterilization at 121 °C for 50 minutes. After cooling to room temperature, solid cultures of *Ganoderma lucidum*, *Lentinula edodes*, and *Pleurotus ostreatus* (accounting for 20% of the substrate mass) were inoculated into the medium under aseptic conditions. The inoculated mixture was then transferred into molds, covered with plastic wrap, and perforated with a fine needle to allow oxygen supply. The molds were subsequently incubated for 7 days in a constant temperature and humidity chamber set at 25 °C and 60% relative humidity. Figures S1–S3 respectively show the mycelial growth after 7 days of cultivation on seven types of carbon source substrates—rice straw, soybean straw, corn cobs, corn stalks, sand willow, poplar wood chips, and peanut straw (from left to right).

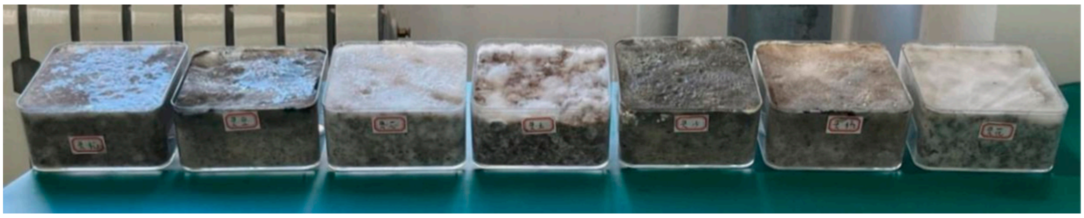

Figure S1. Growth of *Ganoderma lucidum* mycelium on different carbon sources

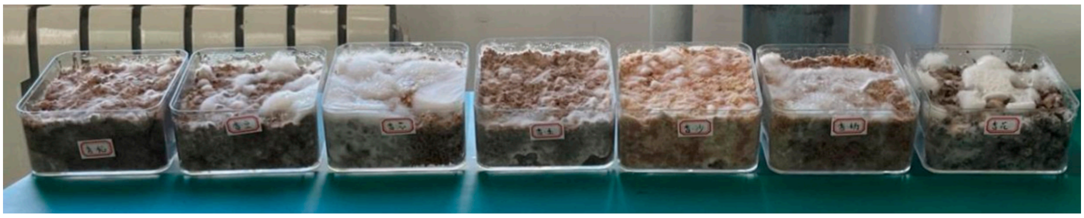

Figure S2. Growth of *Lentinula edodes* mycelium on different carbon sources

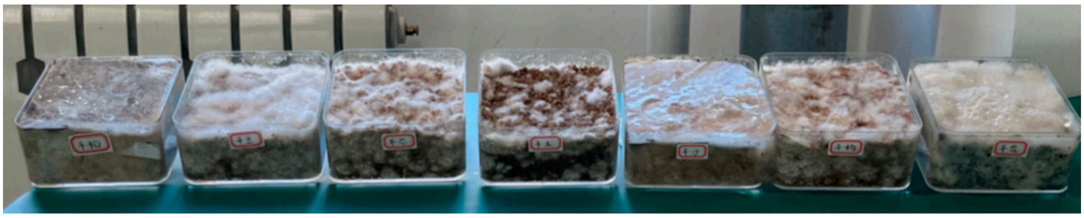

Figure S3. Growth of *Pleurotus ostreatus* mycelium on different carbon sources

Observations indicate that the three fungi exhibit distinct mycelial growth characteristics, particularly in terms of growth rates. *Ganoderma lucidum* mycelium grows the fastest, followed by *Pleurotus ostreatus*, while Lentinula edodes mycelium grows the slowest. Figure S1 shows that substantial mold growth was observed on all substrates inoculated with *Ganoderma lucidum* mycelium, except on those containing peanut straw, corn cobs, and corn stalks. On the peanut straw substrate, *Ganoderma lucidum* mycelium grew most densely, covering the entire surface of the medium. Figure S3 shows that *Pleurotus ostreatus* mycelium grew rapidly on both the Salix psammophila and peanut straw substrates, with no mold growth observed. Considering all factors, peanut straw was selected as the carbon source for *Ganoderma lucidum*, while Salix psammophila was chosen as the carbon source for *Pleurotus ostreatus*.

2 Nitrogen source adaptation

After determining the carbon source, a substrate formulation for *Pleurotus ostreatus* was prepared using Salix psammophila, a nitrogen source, gypsum, starch, and water in a mass ratio of 70:20:2:8:110, while for *Ganoderma lucidum* a formulation was prepared using peanut straw, a nitrogen source, gypsum, starch, and water in the same ratio. To investigate the effect of different nitrogen sources on mycelial growth, bran powder, cow dung powder, and soybean meal powder were added separately, and the cultures were incubated for 5 days under identical conditions. Figures S4 and S5 display the mycelial growth after 5 days of cultivation on three nitrogen-source substrates—bran powder, cow dung powder, and soybean meal powder (from left to right). Figure S4 shows that *Ganoderma lucidum* exhibited numerous black spots, low mycelial density, and restricted growth on the soybean meal and cow dung media. In contrast, Figure S5 indicates that *Pleurotus ostreatus* formed large mycelial patches and showed extensive mycelial spread on the cow dung medium, suggesting that *Pleurotus ostreatus* is more suitable for cow dung as a nitrogen source. Therefore, bran was selected as the nitrogen source for *Ganoderma lucidum*, while cow dung was chosen for *Pleurotus ostreatus*.

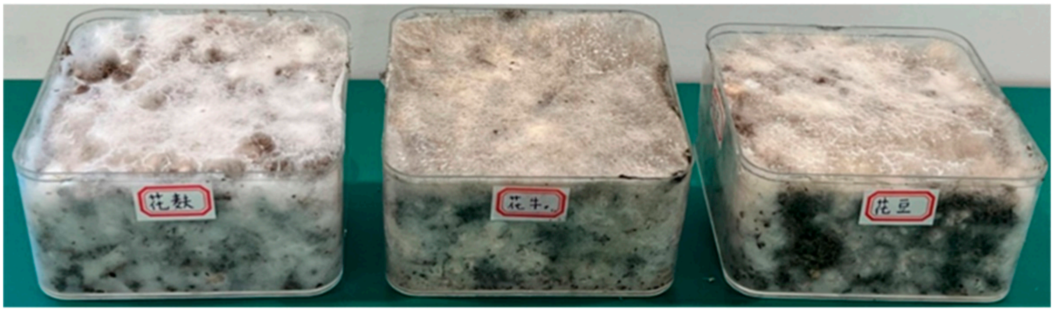

Figure S4. Growth of *Ganoderma lucidum* mycelium on different nitrogen sources

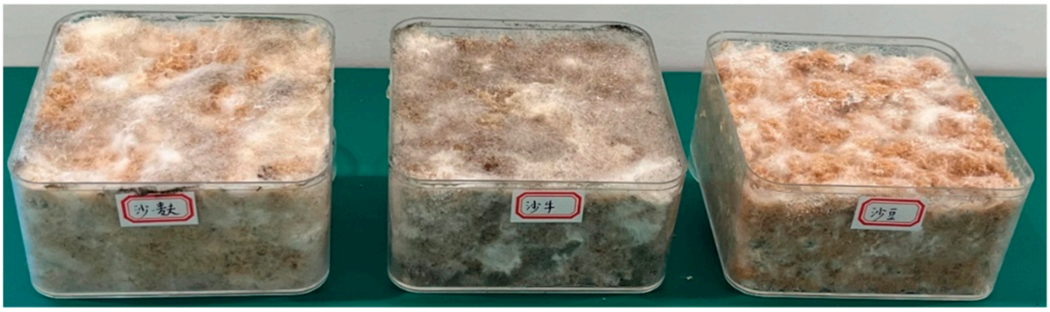

Figure S5. Growth of *Pleurotus ostreatus* mycelium on different nitrogen sources

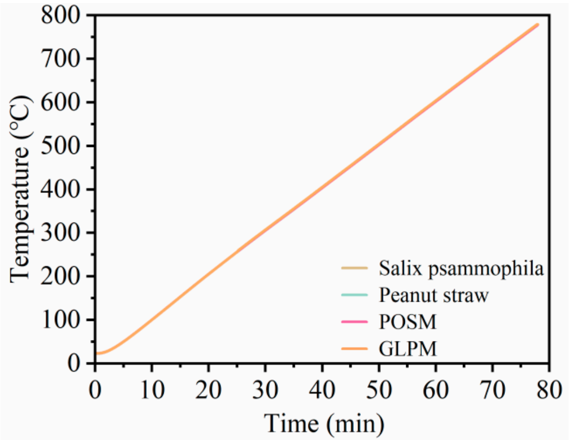

Figure S6. Temperature–time profile during thermogravimetric analysis (TGA)

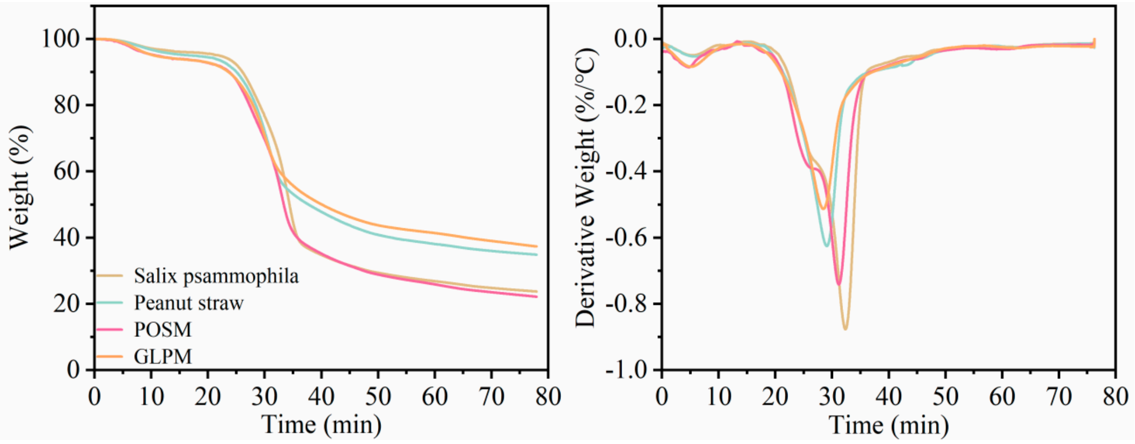

Figure S7. TG and DTG curves as a function of temperature
